# Mesenchymal Stem Cells Increase Neo-Angiogenesis and Albumin Production in a Liver Tissue-Engineered Engraftment

**DOI:** 10.3390/ijms17030374

**Published:** 2016-03-12

**Authors:** Amedeo Carraro, Maurizio Buggio, Chiara Gardin, Umberto Tedeschi, Letizia Ferroni, Barbara Zavan

**Affiliations:** 1 Department of General Surgery and Odontoiatrics, Liver Transplant Unit, University Hospital of Verona, P.le Aristide Stefani 1, Verona 37126, Italy; amedeo.carraro@ospedaleuniverona.it (A.C.); umberto.tedeschi@ospedaleuniverona.it (U.T.); 2 Nanomedicine Lab, Institute of Inflammation and Repair, Faculty of Medical & Human Sciences, University of Manchester, Manchester M13 9PT, UK; maurizio.buggio@manchester.ac.uk; 3 Department of Biomedical Sciences, University of Padova, Viale Giuseppe Colombo, 3, Padova 35131, Italy; letizia.ferroni@unipd.it

**Keywords:** mesenchymal stem cell, hepatocyte, angiogenesis, hyaluronan-based scaffold, engineered liver tissue

## Abstract

The construction of a three-dimensional (3D) liver tissue is limited by many factors; one of them is the lack of vascularization inside the tissue-engineered construct. An engineered liver pocket-scaffold able to increase neo-angiogenesis *in vivo* could be a solution to overcome these limitations. In this work, a hyaluronan (HA)-based scaffold enriched with human mesenchymal stem cells (hMSCs) and rat hepatocytes was pre-conditioned in a bioreactor system, then implanted into the liver of rats. Angiogenesis and hepatocyte metabolic functions were monitored. The formation of a *de novo* vascular network within the HA-based scaffold, as well as an improvement in albumin production by the implanted hepatocytes, were detected. The presence of hMSCs in the HA-scaffold increased the concentration of growth factors promoting angiogenesis inside the graft. This event ensured a high blood vessel density, coupled with a support to metabolic functions of hepatocytes. All together, these results highlight the important role played by stem cells in liver tissue-engineered engraftment.

## 1. Introduction

Liver transplantation still represents the only effective treatment for patients with liver failure. Unfortunately, the increasing demand for organs is so great that many patients die while awaiting transplantation [1]. In order to deal with this problem, many researchers have attempted to develop different extracorporeal bioartificial liver systems to provide a temporary support for exhausted liver; these are cell-based life support devices intended to enhance hepatic detoxification and protein synthesis functions in order to compensate for a whole liver [2,3,4,5]. However, such supports are limited and can only temporarily replace a subset of essential hepatic functions, suggesting the necessity to recapitulate all liver function by alternative approaches. *De novo* fabrication of three-dimensional (3D) liver tissue represents a potential strategy to cope with this problem and the shortage of donor organs; the existing results in tissue engineering emphasize the promising strategy to create, in the future, organs for transplantation. However, liver has a complex and defined organization in which hepatocytes and non-parenchymal cells are precisely arranged, and vascular and biliary networks are interdigitated in a highly-aligned micro-architecture. In front of these aspects and the limitation related to the marked specialization of these cells, liver reconstruction is still limited by many factors; first of all, the lack to recapitulate an adequate vasculature, which is able to maintain viable and functional cells outside the natural liver environment [6,7]. Based on the fundamental role of environmental factors, translational research has directed many efforts to study the extracellular matrix (ECM) and restore its potential influences on cell survival and function [8,9]. A suitable perfusion of metabolically-active tissue requires intimate localization of cells to a dense vasculature in a highly organized manner [10,11]. According these considerations, we have examined recent advances in the field of stem cell and nanotechnology research in order to produce a vascularized and functional tissue-engineered liver graft [12,13,14,15,16]. Advanced tissue engineering strategies were applied through the pre-conditioning of cell-seeded 3D scaffolds in a bioreactor system, followed by their implantation in an animal model. In particular, a hyaluronan (HA)-based scaffold was used as biodegradable platform to study the behavior of human mesenchymal stem cells (hMSCs) co-cultured with hepatocytes.

## 2. Results and Discussion

### 2.1. Three-Dimensional (3D) Culture Preparation

**Figure 1:**
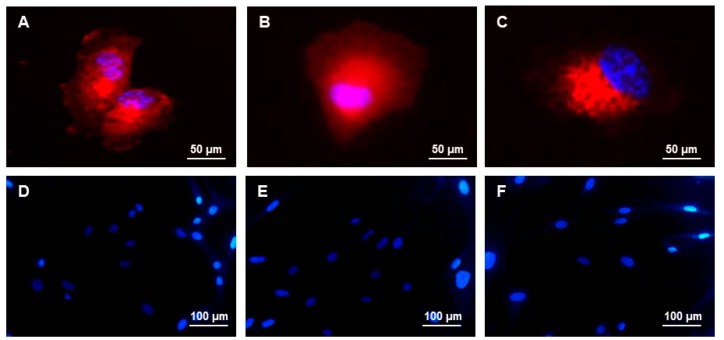
Immunofluorescence staining in human mesenchymal stem cells (hMSCs) at passage three. hMSCs were positive (red) for the stemness markers: (**A**) CD73; (**B**) CD90; and (**C**) CD105. hMSCs were negative for (**D**) fibroblast; (**E**) CD34, and (**F**) CD31 Cell nuclei (blue) were stained with Hoechst 33342.

As reported in the Experimental Section, we have isolated rat hepatocytes and hMSCs from adipose tissue, then amplified them up to passage three. According to the International Society for Cellular Therapy criteria, hMSCs are plastic-adherent cells, expressing CD73, CD90, and CD105, lack CD34 antigens, and have a trilineage mesenchymal differentiation [17]. The phenotypic characterization by immunofluorescence (IF) revealed that our hMSCs at passage three were positive for CD73, CD90, and CD105 markers (Figure 1A–C), and negative for fibroblast marker and CD 34 (Figure 1D,E). Cells were also negative for markers related to endothelial phenotype, such as CD31 (Figure 1F).

**Figure 2:**
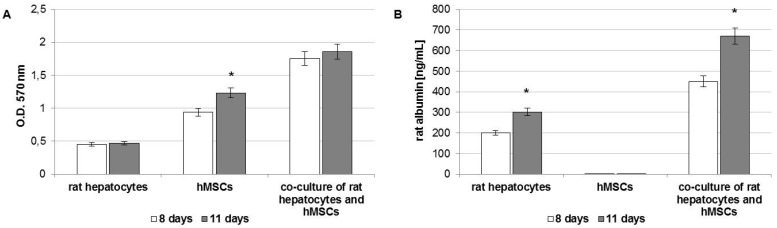
(**A**) Methyl thiazolyl-tetrazolium (MTT) assay; (**B**) albumin enzyme-linked immunosorbent assay (ELISA) assay on three-dimensional (3D) cultures of rat hepatocytes, hMSCs, and rat hepatocytes with hMSCs (ratio 1:1) after 8 and 11 days of culture in the bioreactor system. Data are presented as the mean ± standard deviation (*n* = 3 per group). * *p* value ≤ 0.05.

After harvesting by trypsin treatment, the cells were seeded onto HA-based scaffold tubes in order to prepare three different combinations of 3D cultures: mono-culture of rat hepatocytes, mono-culture of hMSCs, and co-culture of rat hepatocytes and hMSCs (ratio 1:1). All cell-seeded scaffolds were maintained at temperature (37 °C) and carbon dioxide (5%) controlled up to four days. Half of the cell-seeded scaffolds were maintained *in vitro* condition for seven more days, whereas the other half were implanted in rat models for seven days. The 3D cultures maintained *in vitro* up to 11 days were analyzed through methyl thiazolyl-tetrazolium (MTT) and albumin enzyme-linked immunosorbent assay (ELISA) assays at eight and eleven days from cell seeding (Figure 2).

The viability of the three 3D cultures was quantified by means of the MTT assay (Figure 2A). An adequate cellular survival was demonstrated in all culture conditions at eight days. More detailed analyses evidenced that there was no detectable increase in MTT values over time in the mono-culture of rat hepatocytes. A significant increase over time in cell proliferation was observed in scaffolds containing hMSC alone, whereas a slight increase was detected in the co-culture of rat hepatocytes and hMSCs.

The cellular functionality of 3D cultures was evaluated by quantification of the secreted albumin in culture media through an ELISA assay (Figure 2B). The albumin secretion was observed only in mono-culture of rat hepatocytes and in the co-culture of rat hepatocytes and hMSCs. In particular, higher values in albumin secretion were recorded when hepatocytes were co-cultured with hMSCs. This preliminary *in vitro* result suggests that hMSCs could have the ability to support and influence the albumin synthesis of hepatocytes.

### 2.2. In Vivo Experiments

**Figure 3:**
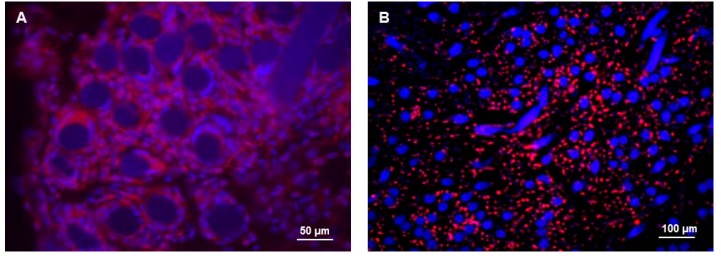
Immunofluorescence staining in hMSCs (**A**) before and (**B**) after implantation in a rat model. hMSCs in the scaffold interstices are identified by red positivity for CD73. Hyaluronan (HA) fibers of the scaffold are blue.

After four days of *in vitro* cultures, the three 3D cultures were implanted in rat models. The pocket-scaffolds were explanted from animals after seven days, then molecular and morphological analyses were performed. The presence of hMSCs has been evaluated before and after the *in vivo* implantation by IF staining of CD73 (Figure 3). After four days of *in vitro* culture, hMSCs are homogeneously distributed (red stain) around the HA fibers of the scaffold (blue stain) (Figure 3A). After seven days of *in vivo* implantation, hMSCs are still present and retained in the scaffold, even if decreased (Figure 3B).

**Figure 4:**
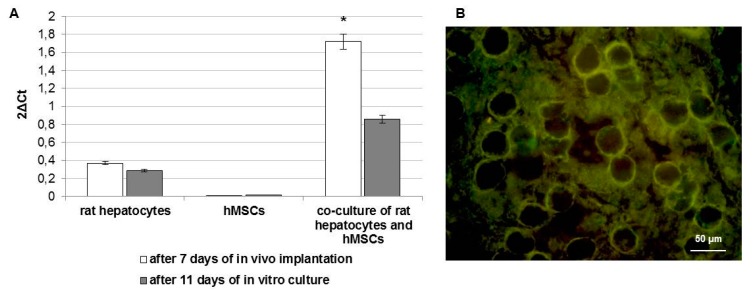
(**A**) Rat albumin mRNA in pocket-scaffolds derived from 3D mono-culture of rat hepatocytes, 3D mono-culture of hMSCs, or 3D co-culture of rat hepatocytes and hMSCs at day seven from implantation in a rat model (white bars) and at day 11 of *in vitro* cultures (gray bars). Results are related to nine rats that have received the three different experimental settings. Data presented as the mean ± standard error of three measurements. * *p* value ≤ 0.05; (**B**) IF staining of rat albumin (green) in pocket-scaffold (black) enriched with rat hepatocytes and hMSCs at seven days from implantation in a rat model.

The rat albumin production related to cells present in the pocket-scaffolds was detected by real-time PCR and IF staining (Figure 4). Gene expression profile revealed albumin mRNA in pocket-scaffolds derived from 3D mono-culture of rat hepatocytes and 3D co-culture of rat hepatocytes and hMSCs (Figure 4A). Results related to the album production *in vivo* (white bars) are compared with those obtained by the correspondent cultures *in vitro* after the same days (namely, after 11 days of *in vitro* cultures; gray bars). This finding confirmed that high level of albumin is reached only when hMSCs are present in the 3D scaffold. IF staining on pocket-scaffolds enriched with rat hepatocytes and hMSCs also confirmed the presence of rat albumin (green) around the HA fibers (black) of the scaffold (Figure 4B).

**Figure 5:**
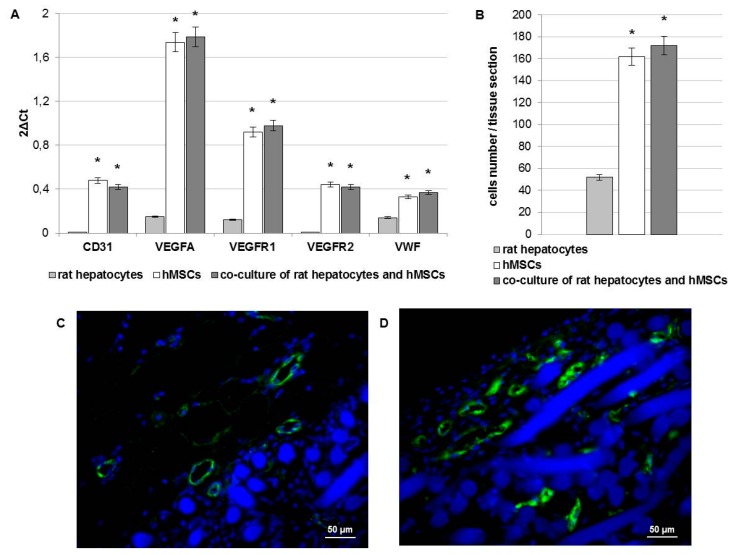
(**A**) Gene expression profiles of endothelial markers in pocket-scaffolds derived from 3D mono-culture of rat hepatocytes, 3D mono-culture of hMSCs, and 3D co-culture of rat hepatocytes and hMSCs at seven days from implantation in a rat model. VEGFA: vascular endothelial growth factor A; VEGFR1: vascular endothelial growth factor receptor 1; VEGFR2: vascular endothelial growth factor receptor 2; VWF: von Willebrand factor. Results are related to nine rats that have received the three different experimental set. Data presented as the mean ± standard error of three measurements. * *p* value ≤ 0.05.; (**B**) Semi-quantitative analysis of cells positive for VWF in pocket-scaffolds after seven days of *in vivo* implantation. Data are presented as the mean ± standard deviation (*n* = 3 per group). * *p* value ≤ 0.05; (**C**, **D**) Immunofluorescence staining of VWF (green) in pocket-scaffold enriched with rat hepatocytes and hMSCs displaying VWF positivity both in the edge and within the pocket-scaffold. Nuclei (blue) stained with Hoechst.

In addition, the presence of blood vessels in the pocket-scaffolds were investigated by gene expression and IF staining of endothelial markers (Figure 5). Gene expression analysis reported in Figure 5A shows the mRNA levels of the endothelial markers CD31 (alias PECAM1, platelet endothelial cell adhesion molecule), vascular endothelial growth factor A (VEGFA), vascular endothelial growth factor receptor 1 (VEGFR1 alias FLT1), vascular endothelial growth factor receptor 2 (VEGFR2 alias KDR), and von Willebrand factor (VWF). These endothelial markers were mainly detected in pocket-scaffolds consisting of hMSCs alone or in association with hepatocytes. The early signs of angiogenesis were revealed also by IF staining of VWF. A semi-quantitative analysis of positive cells for VWF in pocket-scaffolds derived from 3D mono-culture of rat hepatocytes, 3D mono-culture of hMSCs, or 3D co-culture of rat hepatocytes and hMSCs was performed (Figure 5B). The counting of cells positive for VWF revealed an increase in the number of endothelial cells when the pocket-scaffolds were enriched by hMSCs. Contextually, a more detailed observation of pocket-scaffolds structure after seven days of *in vivo* implantation highlighted the presence of high vessel density areas both in the edge (Figure 5C) and within (Figure 5D) the pocket-scaffold. This large increase of neo-vascularization with the use of hMSCs on a HA-based scaffolds represents, in our opinion, a result of particular interest. Based on the fact that hMSCs are CD34-negative cells and are known to promote activation of endogenous cells through a paracrine activity, we suppose that our stem cell population might have activated host endothelial progenitor cells; on the other hand, trans-differentiation events cannot be excluded.

The morphological analyses demonstrated that in pocket-scaffolds consisting of rat hepatocytes and hMSCs the formation of new vessels occurred in the edge of the scaffold; furthermore, a significant migration of neo-developed vessels extended centripetally from the border towards the center of the matrix. These findings were supported by the molecular analyses that revealed a significant increase in the expression of endothelial markers, such as CD31, VEGFA and its receptors, and VWF. Generally, the formation of new capillaries is initiated by growth factors [18]; however, in our model, the HA-based scaffold has fully mimicked the native ECM which allowed the capillaries of surrounding native omentum to promote the engraftment of the engineered tissue. Unfortunately the vascularization of grafts is often inadequate, reducing the supply of oxygen and nutrients, as well as decreasing the degradation of waste products, which directly affects the survival of cells within the graft [19]. Interestingly, our results demonstrated that the pocket-scaffolds enriched with hMSCs have ensured the metabolic function of the rat hepatocytes, which were able to produce albumin.

**Figure 6:**
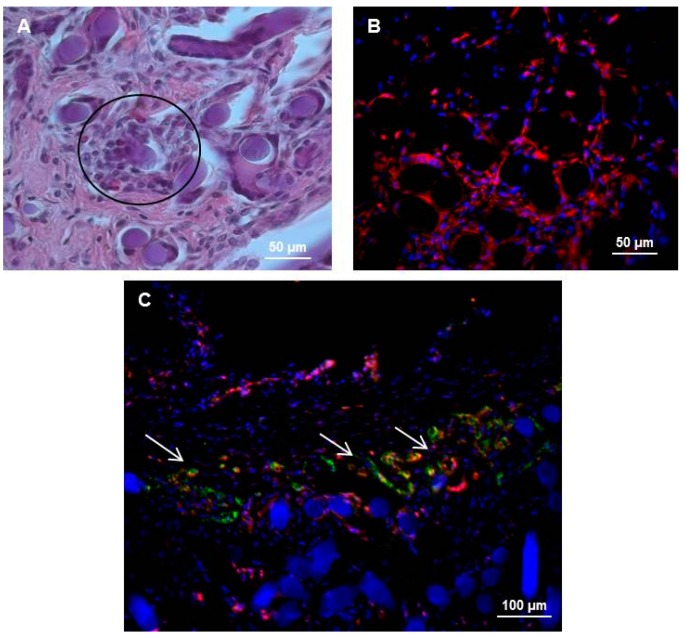
(**A**) The hematoxylin and eosin (H&E) staining of pocket-scaffold enriched with rat hepatocytes and hMSCs showing the presence of hepatocytes (black circle) organized into clusters inside the HA fibers of the pocket-scaffold; (**B**) Immunofluorescence (IF) staining of Vimentin (red) in pocket-scaffold enriched with rat hepatocytes and hMSCs revealing cytoskeleton of mesenchymal cells. Nuclei (blue) stained with Hoechst; (**C**) Double IF staining showing VWF-positive endothelial cells (green) and CD73-positive hMSCs (red) inside the scaffold. Cell nuclei and HA fibers are blue. Vessels are indicated by white arrows.

The morphology of rat hepatocytes and hMSCs within the pocket-scaffolds was also analyzed. The hematoxylin and eosin (H&E) staining in Figure 6A shows the rat hepatocytes (black circle) clearly organized into clusters inside the HA fibers of the pocket-scaffold. They retained their characteristic phenotype with a hexagonal shape often associated with double nuclei. Additionally, the staining of Vimentin (red in Figure 6B), the major cytoskeletal component of mesenchymal cells [20], highlighted the persistence of hMSCs outlining their morphology and adhesion on the HA-based scaffold (black). Furthermore, through a double IF staining against VWF and CD73, the structure of the vessels in the pocket-scaffold was analyzed. In Figure 6C, hMSCs positive to CD73 (red) appear closely associated to VWF-positive cells (green). This observation suggests that hMSCs could drive and promote the formation of new blood vessels inside the scaffold.

According with these results, we demonstrate that the presence of hMSCs within the pocket-scaffold is fundamental to guarantee its engraftment. Indeed, hMSCs assured a better and efficient modulation of factors involved in neo-angiogenesis, adhesion, migration, proliferation, and differentiation of endothelial cells [21,22]. We hypothesize that hMSCs of the pocket-scaffold promoted the neo-angiogenesis by increasing the migration of cells from the surrounding host tissue, and by supporting the proliferation and differentiation of endothelial cells. Promoting the formation of blood vessels, hMSCs also supported hepatocyte vitality and metabolic function, such as albumin production. This was confirmed by a higher metabolic activity of hepatocytes observed only when the pocket-scaffold was enriched with hMSCs. It is proposed that hMSCs can play a key role in releasing trophic factors, which are important in cell protection and support following injury [17,23,24,25,26,27,28]. In our previous *in vitro* study, we have already demonstrated that hMSCs support hepatocyte maturation in a bioreactor system [29]. In this work, we confirmed that hMSCs support hepatocyte maturation also in an *in vivo* engraftment, and that the HA-based scaffold plays a critical role in promoting the proliferation of endothelial cells, especially in the area surrounding the scaffold.

## 3. Experimental Section

### 3.1. Biomaterial

In this study, a material derived from the total esterification of HA with benzyl alcohol (HYAFF-11™, Fidia Advanced Biopolymers, Abano Terme, Padova, Italy) has been used. The product is a no-crosslinked linear polymer of unknown molecular weight, that is insoluble in aqueous solution but hydrolyzes spontaneously over time, releasing benzyl alcohol and HA. The properties of these substrates are previously described in detail by Vindigni *et al.* [30]. HYAFF-11™ was used in flexible non-woven meshes (50 μm-thick fibers, specific weight of 100 g/m^2^) creating a final tube-shape.

### 3.2. Animals

Livers of eight male Lewis rats weighing 200 to 350 g were used for hepatocyte harvesting. In addition, nine male athymic nude rats weighing 250 to 400 g undergone surgery for testing cultured scaffolds. The animals were obtained from Charles River Laboratories (Wilmington, MA, USA); they were maintained on a 12 h-light-dark cycle and given rat chow and water *ad libitum*. All procedures were approved by the Institutional Animal Care and Use Committee of the University of Padova (Padova, Italy, 9 April 2009; 21370), and performed according to the National Institutes of Health Guidelines for the care and use of laboratory animals.

### 3.3. Cells

#### 3.3.1. hMSC Isolation from Adipose Tissue

hMSCs were extracted from adipose tissues of five healthy women and five healthy men (age ranging from 21 to 36 years old; body mass index (BMI) ranging from 30 to 38) undergoing cosmetic surgery procedures, according to the guidelines of the plastic surgery clinic at the University of Padova. Written informed consent was obtained from all subjects, in accordance with the Helsinki Declaration. The Ethical Committee of Padova Hospital approved the research protocol (5 March 2009; 20150). The adipose tissues were digested and the cells isolated and expanded as previously described [31]. Briefly, the adipose tissue was washed with phosphate-buffered saline (PBS, EuroClone, Milan, Italy) and digested using a solution of 0.075% collagenase from Clostridium histolyticum type II (Sigma-Aldrich, St. Louis, MO, USA) in Hank’s balanced salt solution (HBSS, Lonza S.r.l., Milano, Italy), for 3 h at room temperature and under slow agitation. At the end of the digestion, the collagenase activity was blocked with an equal volume of cDMEM, which consisted of Dulbecco’s modified Eagle’s medium (DMEM, Lonza S.r.l.) supplemented with 10% fetal bovine serum (FBS, Bidachem S.p.A., Milano, Italy) and 1% Penicillin/ Streptomycin (P/S, EuroClone). After centrifugation for 4 min at 1200 rpm, the pellet was washed in PBS and filtered with a sterile 70 μm cell strainer (BD Biosciences, Mississauga, ON, Canada). The cells were transferred to a 25 cm^2^ tissue culture flask in cDMEM and incubated at 37 °C and 5% CO_2_. After three days, floating cells were discarded and fresh medium was added on the adherent cells.

#### 3.3.2. Hepatocytes Isolation from Rat Liver

Rat hepatocytes were isolated from livers of eight male Lewis rats, weighing 200 to 350 g. Liver tissue underwent collagenase digestion as previously described by Seglen and coworkers [32]. Briefly, liver tissues were isolated and perfused with 50 mL of calcium-free PBS (EuroClone), warmed to 37 °C prior to use. Then, liver tissues were digested in a mix of trypsin (EuroClone) and collagenase (Sigma-Aldrich) at 37 °C. This resulted in blanching, softening, and dissociation of hepatic tissue and provided complete digestion of the liver in 20 min. Hepatocytes were released by mincing and pipetting with a large-bore pipette. The cell suspension was filtered through a sterile 100 μm cell strainer (BD Biosciences) into a tube placed on ice, and centrifuged at 1200 rpm for 5 min. The cells were washed two times in cold PBS and transferred to a 25 cm^2^ tissue culture flask. The hepatocytes were cultured in DMEM–Ham’s F12 (EuroClone) supplemented with 1% Glutamine (EuroClone), 1% P/S, and 10% FBS.

### 3.4. 3D Culture Preparation

Three different 3D cultures were prepared by seeding cells onto HA-based tubes: (a) mono-culture of rat hepatocytes; (b) mono-culture of hMSCs; and (c) co-culture of rat hepatocytes and hMSCs (ratio 1:1).

Each HA-based tube was seeded with 1 × 10^6^ viable cells, except for culture (c) (in this case: 1 × 10^6^ cells each amounting to 2 × 10^6^ cells), previously mixed with 500 µL of fibrin glue (Baxter AG, Vienna, Austria). All 3D cultures were maintained with DMEM supplemented with 10% FBS, 1% glutamine, and 10 nM dexamethasone in a bioreactor at 37 °C and 5% CO_2_. The bioreactor consisted of eight independent chambers, as previously described [29]. The seeded HA-based tubes were placed in the bioreactor, one in each separate chamber, where a perfusion medium flow rate of 0.5 mL/min through each scaffold was assured.

All cell-seeded scaffolds were maintained in the bioreactor up to four days. At this point, half of the scaffolds were implanted *in vivo* in nine rats, whereas the other half was left in the bioreactor for other seven days.

### 3.5. Methyl Thiazolyl-Tetrazolium (MTT) Assay

To determine the viability of cells grown on HA-based tubes, the MTT-based cytotoxicity assay was performed according to the method of Denizot and Lang with minor modifications [33]. The test is based on mitochondria viability, *i.e.*, only functional mitochondria can oxidize an MTT solution, giving a typical blue-violet end product. After harvesting the culture medium, the cells were incubated for 3 h at 37 °C in 1 mL of 0.5 mg/mL MTT solution prepared in PBS. After removal of the MTT solution by pipette, 0.5 mL of 10% dimethyl sulfoxide in isopropanol was added for 30 min at 37 °C. For each sample, absorbance values at 570 nm were recorded in duplicate on 200 μL aliquots deposited in 96-well plates using a multilabel plate reader (Victor 3, Perkin Elmer, Milano, Italy). 3D cultures were examined at eight and 11 days from the seeding.

### 3.6. Albumin Enzyme-Linked Immunosorbent Assay (ELISA) Assay

Albumin secreted by the cells was quantified using the AssayMax Rat Albumin ELISA Kit (Gentaur, Brussels, Belgium). This assay employs a quantitative sandwich enzyme immunoassay technique that measures rat albumin in cell culture medium. A polyclonal antibody specific for rat albumin has been pre-coated onto a 96-well microplate. Albumin in standards and culture media was sandwiched by the immobilized polyclonal antibody and biotinylated polyclonal antibody specific for rat albumin, which was recognized by a streptavidin-peroxidase conjugate. All unbound material was then washed away and a peroxidase enzyme substrate was added. The color development was measured on a microplate reader (Victor 3, Perkin Elmer) at a wavelength of 450 nm.

Cell culture media were harvested from the circulating medium in bioreactor at eight and 11 days, then frozen at −20 °C until the analysis. The minimum detectable dose of albumin with this kit is typically 0.7 ng/mL.

### 3.7. 3D Culture Surgical Implantation

The scaffolds, three for each type of 3D cultures, were implanted in nine athymic rats up to seven days. Rats were subjected to surgery under isoflurane anesthesia and allowed to recover after surgical procedure. Implantation was obtained by making small mid-line abdominal incision, and scaffold implantation was performed with externalized omentum which was gently wrapped around the seeded HA-based tube, constituting a final pocket structure. All operations were performed under sterile conditions.

### 3.8. Hematoxylin and Eosin (H&E) and Immunofluorescence (IF) Stainings

The pocket-scaffolds were fixed in 4% phosphate-buffered formalin pH 7, embedded in paraffin, then cut into 7 μm thick-sections.

For H&E staining, the sections were stained with the nuclear dye hematoxylin (Sigma-Aldrich), and the counterstain eosin (Sigma-Aldrich). For IF staining, the sections were incubated in 2% bovine serum albumin (BSA, Sigma-Aldrich) solution in PBS for 30 min at room temperature. The sections were then incubated with the primary antibodies in 2% BSA solution in a humidified chamber overnight at 4 °C. The following primary antibodies were used: anti-human CD73 (Abcam, Cambridge, UK), anti-human CD90 (Abcam), anti-human CD105 (Santa Cruz Biotechnology Inc., Paso Robles, CA, USA), anti-human CD34 (Abcam), anti-fibroblast (Abcam), anti-human CD31 (Abcam), rabbit anti-rat Albumin antibody 1:100 (Bethyl Laboratories, Inc., Montgomery, TX, USA), rabbit anti-Von Willebrand factor antibody 1:100 (Dako, Milan, Italy), and mouse anti-Vimentin antibody 1:100 (Abcam, Cambridge, UK). IF staining was performed with secondary antibodies: anti-rabbit IgG DyLight 488 labeled (KPL, Gaithersburg, MD, USA), and anti-mouse IgG DyLight 549 labeled (KPL) in 2% BSA for 1 h at room temperature. Nuclear staining was performed with 2 μg/mL Hoechst H33342 (Sigma-Aldrich) solution for 5 min. The sections were coverslipped with a drop of mounting medium.

### 3.9. Real-Time PCR

Total RNA was extracted from each sample by using the TRIzol^®^ Reagent (Invitrogen, Carlsbad, CA, USA). The samples were quantified using the NanoDrop spectrophotometer (NanoDrop™ 1000, Thermo Scientific, Waltham, MA, USA). For the first-strand cDNA synthesis, 500 ng of total RNA was reverse transcribed using M-MLV RT (Moloney Murine Leukemia Virus Reverse Transcriptase, Invitrogen, Paisley, UK) according to the manufacturer’s protocol. Primers were selected for each target gene with Primer 3 software. Real-time PCRs were carried out using the designed primers at a concentration of 300 nM and FastStart SYBR Green Master (Roche Diagnostics, Mannheim, Germany) on a Rotor-Gene 3000 (Corbett Research, Sydney, Australia). Thermal cycling conditions were as follows: 15 min denaturation at 95 °C; followed by 40 cycles of 15 s denaturation at 95 °C; annealing for 30 s at 60 °C; and 20 s elongation at 72 °C. Values were normalized to the expression of the β-actin internal reference, whose abundance did not change under our experimental conditions. Experiments were performed with three different cell preparations and repeated at least three times.

### 3.10. Semi-Quantitative Analysis of Cells

In order to detect the presence of endothelial cells in the mesh scaffolds, masked microscopic examinations were performed on immunostained sections. Endothelial cells were identified by VWF-monoclonal antibody immunostaining, as described. Briefly, two investigators analyzed in a masked fashion at least three slides for each experiment by light microscopy using 20× as the initial magnification. Each slide contained three sections of a specimen and five fields of 322 mm^2^ each, were analyzed for each tissue section. Experiments were performed at least three times and values were expressed as the mean ± standard deviation.

### 3.11. Statistical Analysis

Statistical analyses were performed using SPSS software (SPSS Inc., Chicago, IL, USA). The mean values for quantitative data were compared applying non-parametric Kruskal–Wallis test for real-time PCR results. Parametric one-way ANOVA analysis was applied for MTT, ELISA, and semi-quantitative analysis data, following by a *post hoc* Tukey test. *p* value (statistical significance) was set at 0.05.

## 4. Conclusions

The clinical success of an engineered tissue graft is very limited without proper vascularization. To overcome this problem, the stimulation of angiogenesis is the main strategy to ensure the vascularization of engineered grafts. The formation of new blood vessels is facilitated by biomaterials with 3D structures that mimic the native ECM. For this reason, the development of scaffolds equivalent to native ECM represents an important investigation area in the field of vascularized tissue engineering. However, the full restoration of standard vascular function and structural integrity represents a complex aim to achieve because of native ECM complexity and the lack of spatial and temporal integrity of the grafts [34].

In the present work, we have demonstrated that a HA-based scaffold can promote the vascularization of tissue engineered grafts, especially in the border areas. This property must be added to the already known characteristic of low antigenicity, high biodegradability, and good mechanical and hemostatic properties [30,35]. Our results also demonstrate the important role played by hMSCs in neo-angiogenesis and metabolic process. Indeed, hMSCs increased growth factors in the pocket-scaffold and promoted angiogenesis, resulting in a high blood vessel density coupled with a better support to metabolic function of hepatocytes. However, in the current biocompatible and degradable device, the maximum cell mass was physically limited by the mesh surface area of the implantable scaffold. In fact, in our experimental setting, the device was designed to fit within the dimensions of the animal model and was not aimed to show any clinical impact. Both the microfluidics model and the nanotech manufacturing process allow for scalability in higher dimensions to support a larger cell mass for increased hepatic function. Furthermore, ongoing development by our research group will allow the generation of an integrated, larger 3D unit as the basis of a liver assist device to augment function in a clinical setting.

In conclusion, a scaffold with excellent properties enriched with hMSCs could be considered a smart approach to increase the engrafting of engineered tissues. This modification may induce a guided formation of blood vessels through the migration and proliferation of endothelial cells within the graft. In turn, the neo-angiogenesis may guarantee oxygen and nutrients, as well as the elimination of waste products within the graft. Thus, hMSCs seem to support vitality and metabolic functions of hepatocytes creating a proper environment.
